# Plasticity of natural killer cells in pregnant patients infected with SARS-CoV-2 and their neonates during childbirth

**DOI:** 10.3389/fimmu.2022.893450

**Published:** 2022-07-15

**Authors:** Marie Carbonnel, Camille Daclin, Nadine Tarantino, Olivia Groiseau, Véronique Morin, Alice Rousseau, Marc Vasse, Alexandre Hertig, Titouan Kennel, Jean Marc Ayoubi, Vincent Vieillard

**Affiliations:** ^1^ Department of Obstetrics and Gynecology, Hôpital Foch, Suresnes, France; ^2^ University of Versailles, Versailles, France; ^3^ Sorbonne Université, Inserm U1135, CNRS ERL 8255, Centre d’Immunologie et des Maladies Infectieuses (CIMI-Paris), Paris, France; ^4^ Department of Clinical Biology, Hôpital Foch, Suresnes, France; ^5^ INSERM UMRS-1176, University Paris-Sud, Orsay, France; ^6^ Nephrology and Renal Transplantation Department, Hôpital Foch, Suresnes, France; ^7^ Department of Clinic Research, Hôpital Foch, Suresnes, France

**Keywords:** natural killer cells, pregnancy, neonates, SARS-CoV-2, COVID-19

## Abstract

The COVID-19 pandemic has occurred due to infection caused by the SARS-CoV-2 coronavirus, which impacts gestation and pregnancy. In SARS-CoV-2 infection, only very rare cases of vertical transmission have been reported, suggesting that fetal immune imprinting due to a maternal infection is probably a result of changes in maternal immunity. Natural killer (NK) cells are the leading maternal immune cells that act as a natural defense system to fight infections. They also play a pivotal role in the establishment and maintenance of pregnancy. While peripheral NK cells display specific features in patients infected with SARS-CoV-2 in the general population, information remains elusive in pregnant mothers and neonates. In the present study, we analyzed the characteristics of NK cells isolated from both neonatal umbilical cord blood and maternal peripheral blood close to the time of delivery. Phenotype and functions were compared in 18 healthy pregnant women and 34 COVID-19 patients during pregnancy within an ongoing infection (PCR^+^; N = 15) or after recovery (IgG^+^PCR^−^; N = 19). The frequency of NK cells from infected women and their neonates was correlated with the production of inflammatory cytokines in the serum. The expression of NKG2A and NKp30, as well as degranulation of NK cells in pregnant women with ongoing infection, were both negatively correlated to estradiol level. Furthermore, NK cells from the neonates born to infected women were significantly decreased and also correlated to estradiol level. This study highlights the relationship between NK cells, inflammation, and estradiol in patients with ongoing infection, providing new insights into the impact of maternal SARS-CoV-2 infection on the neonate.

## Introduction

Coronavirus disease 2019 (COVID-19), caused by severe acute respiratory syndrome coronavirus 2 (SARS-CoV-2), is a major public health challenge that rapidly spread around the world. Multi-system injuries and death observed in patients are thought to be associated with immune dysfunction induced by the virus (1). Pregnant women are generally more susceptible to viral infection, and their respiratory capacity decreases as the pregnancy progresses (2). Initial reports raised a concern of an excess risk of developing severe COVID-19 for pregnant women infected with SARS-CoV-2, particularly in the third trimester; however, more recent, larger cohorts suggest that only a small fraction of them were severely affected (3). However, COVID-19 induces more maternal and fetal complications during pregnancy, including preeclampsia, intrauterine growth retardation, and preterm birth (3, 4).

During pregnancy, major adaptations occur to protect the mother and her future baby. There is some evidence to support that the maternal immune system weakens during pregnancy, so that it can tolerate the fetus, increasing the risks for certain infections (2). The importance of natural killer (NK) cells in pregnancy is paramount: decidual NK cells play an important role in placentation, remodeling of the spinal arteries, and control of trophoblast invasion (5); peripheral NK cells decrease in number and functionality during pregnancy with a lower rate in the third trimester, which seems to be a useful adaptation to help ensure fetal survival (6). In umbilical cord blood, the number of NK cells is greater than in adults, and these cells are phenotypically and functional immature (7). NK cells are critical effectors of the innate immune response and represent the first line of defense against invading viruses, highlighted by studies on patients with NK cell deficiencies, a condition that leads to the development of fulminant viral infections (8). NK cell activity is shaped by a vast array of receptors, including not only inhibitory receptors, like KIR-L and NKG2A, used to maintain a self-tolerance, but also activating receptors, such as the natural cytotoxicity receptors (NKp30, NKp46), NKG2D, and NKG2C. When these latter signals are activated, NK cells produce an array of proinflammatory cytokines, like IFN-γ and TNF-α, in parallel with the initiation of their cytotoxic functions to specifically eliminate harmful infected host cells (9–11).

A previous clinical study reported that NK cells were markedly decreased in patients with SARS-CoV-2 infection, especially in individuals with severe disease, but restoration was observed after recovery (12, 13). Specifically, NK cells were activated in the peripheral blood of COVID-19 patients and showed high expression of perforin and NKG2C (13). In parallel, it was shown that NK cells were depleted and appeared exhausted, expressing high levels of LAG3 (14). Mechanistically, SARS-CoV-2 can induce NK cell exhaustion *via* Spike 1 protein binding to the HLA-E of lung epithelial cells, thereby triggering the HLA-E/NKG2A pathway (15), suggesting the important role of NK cells in pathological COVID-19 processes. Nevertheless, a better understanding of the role of NK cells in pregnant women infected with SARS-CoV-2 and the consequences on their neonates is urgently needed. To date, it has been shown that vertical transmission is low, i.e. around 5% (3), and preliminary data reveal an increase in NK cells in neonates born after a recent or ongoing COVID-19 infection during pregnancy (16).

The aim of this study was to extensively characterize the phenotypic and functional features of NK cells isolated from pregnant women in cases of ongoing SARS-CoV-2 infection or after recovery, and their neonates, during a period close to the time of delivery.

## Materials and methods

### Participants and data collection

**Figure 1:**
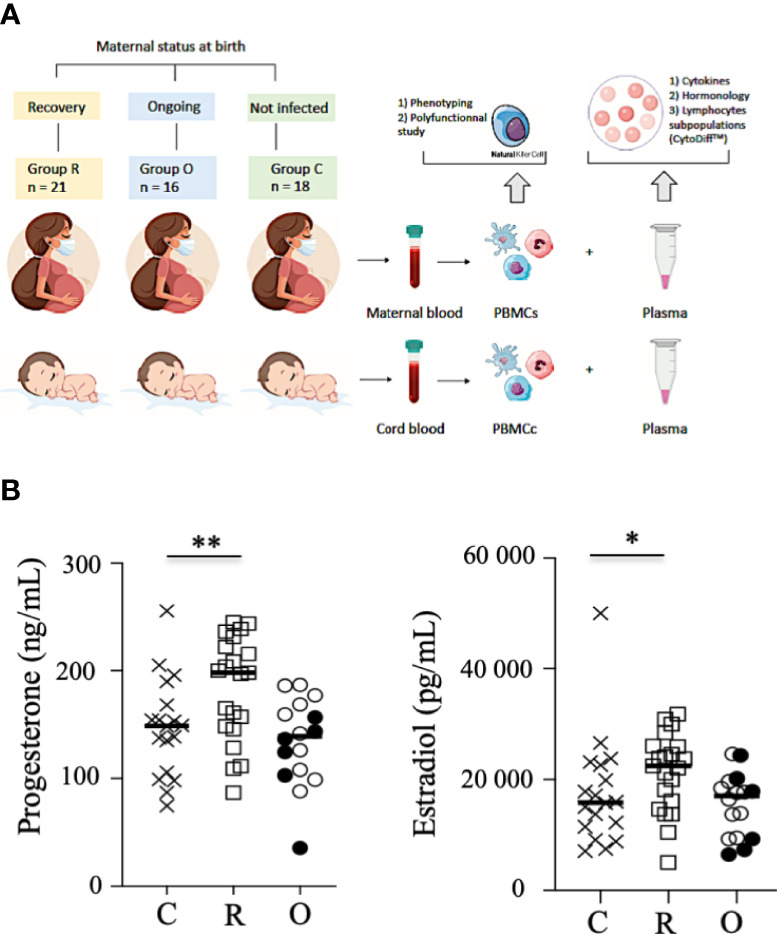
Study outline and hormone production. **(A)**. Study outline of the recruitment of neonates and mothers. **(B)** Production of progesterone and estradiol in the sera of mothers. Data are shown for healthy controls (N= 18; C: crosses), recovered patients (N= 21; R: open squares), and patients with ongoing infection (N=16; O: circles). Open circles represent the asymptomatic patients and closed circles the symptomatic patients. Black lines represent the median. *p < 0.05; **p < 0.001.

We designed the prospective MaterCov cohort study including pregnant women who were infected with COVID-19 and delivered in the Obstetric Department of Foch Hospital (Suresnes, France) (**Table 1**). Between January and July 2021, 37 pregnant women with healthcare insurance infected by SARS-CoV-2 were enrolled in this study (**Figure 1A**). Exclusion criteria were infection with human immunodeficiency virus, toxoplasmosis, rubeola, cytomegalovirus, or syphilis during pregnancy, infection with influenza virus within 10 days prior to delivery, patients under 18 years, and patients under guardianship. The MaterCov study was conducted in accordance with the principles of the Declaration of Helsinki, as well as French statutory and regulatory law, received approval from the Institutional Review Board of Ouest V Rennes in December 2020 (IRB number 2020-A03115-34), and was declared as a clinical trial (No. NCT04726111). Patients received information about the research to be performed on their biological samples and provided written informed consent to participate. Partners also provided consent for the cord blood assays.

**Table 1:** Maternal and neonatal clinical characteristics.

	Ongoing Infection	Recovered	Control	p value
**Maternal characteristics (n)**	**16**	**21**	**18**	
Median age, years (min - max)	34.0 (21 - 40)	31.0 (24 - 43)	32.5 (26 - 44)	0.52
Median BMI (min - max)	28.4 (21 - 36)	25.5 (19 - 31)	27.0 (22 - 34)	0.09
Asthma, number (%)	0	2 (9.5%)	2 (11%)	0.54
Previous HBP^a^, number (%)	0	0	0	NA
Previous Diabetes, number (%)	0	0	0	NA
Gestation (weeks) median (min-max)^b^	38 (30 - 41)	27 (8 - 37)	NA	**<0.001**
Days between SARS-CoV-2^+^ status and birth, median (min - max)^C^	7 (0 - 56)	84 (21 - 225)	NA	**<0.001**
COVID-19 symptoms, number (%)	13 (81%)	18 (86%)	NA	1
Hospitalisation, number (%)	3 (19%)	1(4.8%)	3 (17%)	0.59
Total number of complications (%)	6 (37%)	2 (9.5%)	1 (5.66%)	**0.04**
Stillbirth, number (%)	1 (6.2%)	0	0	0.29
Gestational Diabetes, number (%)	1 (6.2%)	0	0	0.29
HBP, number (%)	0	1 (4.8%)	0	1
Preeclampsia, number (%)	1 (6.2%)	0	0	0.29
Threat of premature delivery, number (%)	1 (6.2%)	0	1 (5.6%)	0.52
IUGR, number (%)	3 (19%)	1 (4.8%)	0	0.10
Term at birth median (min - max)	39 (29 - 41)	40 (37 - 41)	39.5 (37 - 41)	0.68
Pyrexia during labor, number (%)	1 (6.2%)	0	0	0.29
Cesarean section, number (%)	3 (19%)	4 (19%)	1 (5.6%)	0.45
**Neonatal characteristics (n)**	**14**	**19**	**18**	
Female sex, number (%)	7 (50%)	9 (47%)	7 (39%)	0.95
Weight, median (min - max)	3100 (1484 - 4140)	3320 (2760 - 4270)	3515 (2660 - 4198)	0.13
pH, median (min - max)	7.25 (7.20 – 7.35)	7.24 (7.08 – 7.43)	7.23 (7.09 – 7.45)	0.26
Transfer in ICU, number (%)	2 (14.2%)	0	0	0.07
Respiratory distress, number (%)	1 (7.1%)	1 (5.3%)	0	0.75

a Hight blood pressure; ^b^Gestation (weeks) at time of positive SARS-CoV-2^+^ status; ^c^Days between SARS-CoV-2^+^ status (nasopharyngeal swab) and birth. Bold values, significant values.

Patients were categorized according to those with an ongoing infection (N = 16; RT-PCR SARS-CoV-2 positive at delivery) and those who had recovered (N = 21; RT-PCR SARS-CoV-2 negative at delivery, and anti-SARS-CoV-2 IgG serology positive at delivery). The control group was constituted retrospectively, based on the clinical data of infected patients, from 18 non-infected pregnant women without comorbidities such as diabetes, preeclampsia, and high blood pressure (**Table 1**). Importantly, none of the individuals tested (patients and healthy controls) had been vaccinated against SARS-CoV-2. From all individuals, maternal blood and cord blood samples were collected within the 24 h before delivery and at birth, respectively. Hormonology and analysis of lymphocyte subpopulations (CytoDiff™) were performed on all samples by the biology department of Foch Hospital. Peripheral blood mononuclear cells were isolated by standard density centrifugation and then frozen at −150°C, and plasma at −80°C, by the Foch Hospital biological repository resource center.

### Flow cytometry analysis

CD3^−^CD56^+^ NK cells were analyzed in women and neonates for whom peripheral blood mononuclear cells had been frozen. Cells were stained with an appropriate cocktail of monoclonal antibodies (mAbs), described in **Supplementary Table 1**. According to flow cytometry guidelines (17), live cells were acquired (DXFlex, Beckman-Coulter; Brea, CA, USA) and then analyzed with FlowJo software version 10 (TreeStar; Ashland, OR, USA) (**Supplementary Figure 1**). A minimum of 100 CD3^−^CD56^+^ NK cells were acquired on a DXFlex flow cytometer and then analyzed with FlowJo version 10.

### NK cell degranulation assays and cellular production of cytokines

Polyfunctional NK cells were studied with degranulation assays and the cellular production of cytokines was assessed, as previously described (18). NK cells were pretreated overnight in the presence of interleukin (IL)-12 (10 ng/mL) and IL-18 (100 ng/mL) to measure the intracellular production of IFN-γ and TNF-α by NK cells or incubated with standard HLA class 1-negative K562 target cells (ATCC CCL243) at an effector : target (E : T) cell ratio of 1 : 1 to measure degranulation in the presence of anti-CD107a mAb (#H4A3; Becton Dickinson) (**Supplementary Table 1**). Cells were incubated for 5 h in the presence of Golgi Stop and Golgi Plug solutions (BD Biosciences; Franklin Lakes, NJ, USA) and then stained with NK cell surface markers. NK cells were thereafter fixed, permeabilized with a Cytofix/Cytoperm kit (Becton Dickinson; Franklin Lakes, NJ, USA), and then intracellularly stained with anti-IFN-γ and anti-TNF-α mAbs (**Supplementary Table 1**), as described previously (18). Only samples containing at least 100 CD3^−^CD56^+^ NK cells were acquired on a DXFlex flow cytometer and then analyzed with FlowJo version 10; consequently, the number of samples may vary depending on the experiments.

### Cytokine profiling

A CorPlex Human Cytokine 10-Plex Panel 1 Array (116-7BF-1-AB; Quanterix, USA) was used to simultaneously profile 10 cytokines (IL-12p70, IL-1β, IL-4, IL-5, IFN-γ, IL-6, IL-8, IL-22, TNF-α, and IL-10) in maternal and neonatal plasma from individuals, as described in **Table 1**. The assay was performed according to the manufacturer’s instructions. The luminescence was read using an SP-X Imaging System (Quanterix). Sensitivity and selectivity are described on the manufacturer’s website (https://www.thermofisher.com/document-connect/document-connect.html?url=https://assets.thermofisher.com/TFS-Assets/LSG/manuals/MAN0017197_HumanCytokineMagnetic10PlexPanel_TDS.pdf).

### Statistical analysis

For clinical and biological data, Microsoft Excel software was used for data recording and analyses were performed using SAS v9.4. For experimental data, statistical analyses were performed with Prism 8.0 software (GraphPad, CA, USA). The quantitative data are described as median values and categorial variables as percentages. The non-parametric Kruskal–Wallis test was used for comparisons between groups. When statistical differences were observed between patients with ongoing infection and controls, a Mann–Whitney test was performed to compare data of symptomatic and asymptomatic ongoing patients. Correlations between variables were calculated using the non-parametric Spearman rank order test. *P* values > 0.05 were considered insignificant.

## Results

### Demographic and clinical features of COVID-19

The demographic and clinical features of COVID-19^+^ patients are summarized in **Table 1**. From a total of 37 pregnant women infected by the SARS-CoV-2, 16 tested positive by real-time polymerase chain reaction (RT-PCR; nasopharyngeal swab); ten were asymptomatic and six patients were diagnosed as having mild or severe (requiring oxygen supplementation) COVID-19. One of the women with severe disease (p34 patient) was hospitalized at 29 weeks of gestation (WG) for oxygen therapy; she had COVID-19 symptoms for 15 days and no associated comorbidities. Blood tests found an increase in CRP (22 mg/L). On the second day of hospitalization, a stillbirth was diagnosed. She delivered a eutrophic fetus (1480 g) on the same day. Placental analysis found placental abruption. The mother had a complete recovery without sequelae of COVID-19.

Term at birth was similar in all groups, i.e. at 39 WG in patients with ongoing infection, 40 WG in recovered patients, and 39.5 WG in controls. No case of preterm delivery was observed except for the stillbirth. Neonates were not RT-PCR tested for SARS-CoV-2; thus, infection status throughout the manuscript refers solely to the mother.

At 1 month after birth, 60% of mothers answered the phone survey. No mothers had sequelae of COVID-19 infection or other complications; two neonates were hospitalized for 3 to 8 days for pulmonary infection not related to COVID-19 (not shown). No cases of infection or additional complications were reported for the neonates.

Progesterone and estradiol were measured at birth in all groups of mothers and were both significantly increased in recovered patients, as compared to patients with ongoing infection and healthy women (**Figure 1B**).

### Phenotypic characteristics of peripheral NK cells from pregnant women and their neonates in COVID-19

The absolute values of neutrophils, CD14^+^ monocytes, CD45^+^ lymphocytes, B lymphocytes, T lymphocytes, and NK subsets were measured using CytoDiff in cord blood and blood from pregnant women infected with SARS-CoV-2 and compared to healthy controls. The absolute values of B and T lymphocytes were similar in healthy and SARS-CoV-2-infected maternal blood samples (**Supplementary Figure 2**). In contrast, the levels of neutrophils and monocytes were significantly decreased in infected women with ongoing infection, as compared to healthy controls (p = 0.0402), and this more significantly in symptomatic patients (black circles) than asymptomatic patients (p = 0.0176) (**Supplementary Figure 3A**). Of note, in cord blood samples the level of the different cell subsets was similar in infected and control samples, as previously described (16, 19).

**Figure 2:**
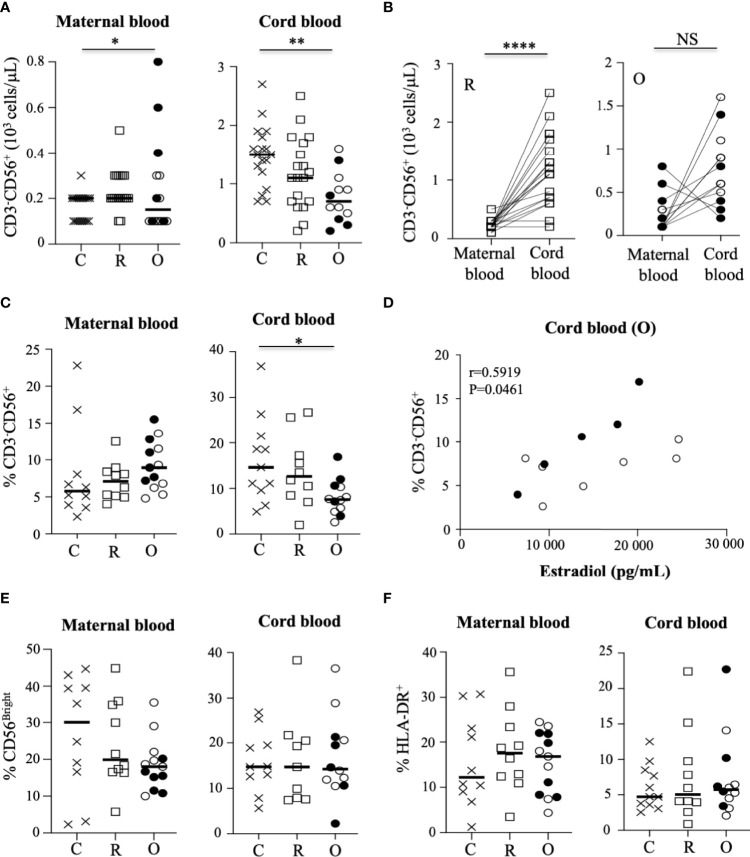
Characteristic of NK cells in mothers and their neonates. **(A)** Absolute values of CD3^-^CD56^+^ NK cells in mothers (maternal blood) and their neonates (cord blood). Data are shown for healthy controls (N= 18; C: crosses), recovered patients (N= 19; R: open squares), and patients with ongoing infection (N=15; O: circles). **(B)** Significant correlation of the absolute values of CD3^-^CD56^+^ NK between mothers (maternal blood) and their neonates (cord blood). for the groups of recovered (R: open squares) and ongoing infection (O: circles). **(C)** Frequency of CD3^-^CD56^+^ NK cells in mothers (maternal blood) and their neonates (cord blood). **(D)** Significant correlation between the frequency of CD3^-^CD56^+^ NK cells in neonates (cord blood) born to mothers with ongoing (O: circles) infection and the production of estradiol in mothers. **(E)** Frequency of CD56^bright^ subpopulation of NK cells gated on CD3^-^CD56^+^ NK cells. **(F)** Frequency of HLA-DR^+^-activated NK cells gated on CD3^-^CD56^+^ NK cells in mothers (maternal blood) and their neonates (cord blood). Frequency’ data are shown for healthy controls (N= 10; C: crosses), recovered patients (N= 10; R: open squares), and patients with ongoing infection (N=13; O: circles). Open circles represent the asymptomatic patients and closed circles the symptomatic patients. Black lines represent the median. *p < 0.05; **p < 0.001; ****p < 0.00001; NS, non significant.

With regard to the NK cell compartment, although some symptomatic patients with ongoing infection had elevated levels of NK cells (closed circles), **Figure 2A** shows that the absolute value of NK cells was similar in patients with ongoing infection, as compared to recovered patients and healthy pregnant women. In contrast, the number of NK cells was significantly decreased in neonates from women with ongoing infection, as compared to other neonates (p = 0.0042) (**Figure 2A**). Of note, a very significant increase in NK cell numbers was observed between healthy women and recovered patients and their neonates (p < 0.0001), but not in patients with ongoing infection (**Figure 2B**). These data are in accordance with those concerning the frequency of NK cells in pregnant women and their neonates (**Figure 2C**), measured by a DXFlex flow cytometer. Importantly, the frequency of NK cells from neonates of mothers with ongoing infection was specifically correlated with the production of estradiol (r = 0.5919; p = 0.0461) (**Figure 2D**), and more specifically if only cord blood samples from symptomatic mothers (closed circles) were tested (**Supplementary Figure 3B**).

Human NK cells can be divided into CD56^bright^ and CD56^dim^ subpopulations, based on the cell surface density of CD56 molecules; these subgroups present distinct phenotypic and functional capacities. Thus, the CD56^dim^ NK cell subset is thought to mediate higher cytotoxic responses, including antibody-dependent cellular cytotoxicity, whereas the CD56^bright^ subset is more involved in immunomodulation, with a higher capacity to produce cytokines like IFN-γ and TNF-α (20). In this study, the frequency of CD56^bright^ and CD56^dim^ cells was similar in the different groups of samples (**Figure 2E** and **Supplementary Figure 4**). In addition, the rate of activated NK cells expressing HLA-DR, reported as a sign of intense chronic activation, was not significantly modulated by SARS-CoV-2 infection in pregnant women and their neonates at birth (**Figure 2F**), contrary to what has been reported for infected patients in the general population (13).

**Figure 3:**
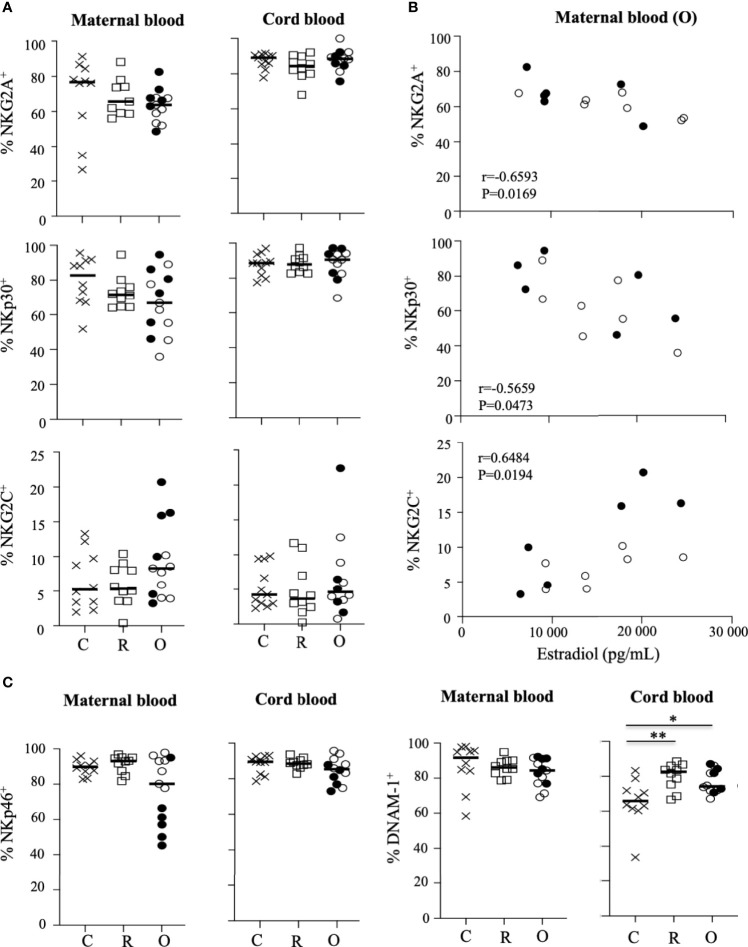
Pattern of receptor expression on CD3^-^CD56^+^ NK cells. **(A)** Frequency of NKG2A, NKG2C, and NKp30 on NK cells gated on CD3^-^CD56^+^ NK cells from mother (maternal blood) and their neonates (cord blood). **(B)** Correlation between the frequency of NK cell receptors and the production of estradiol in mothers (maternal blood) with ongoing infection (O: circles). **(C)** Frequency of NKp46 and DNAM-1 on NK cells gated on CD3^-^CD56^+^ NK cells from mother (maternal blood) and their neonates (cord blood). Data are shown for healthy controls (N= 10; C: crosses), recovered patients (N= 10; R: squares), and patients with ongoing infection (N=13; O: circles). Open circles represent the asymptomatic patients and closed circles the symptomatic patients. Black lines represent the median. *p < 0.05; **p < 0.001.

We next focused our study by analyzing the expression of a large panel of activating and inhibitory receptors on peripheral NK cells from the mothers and their neonates, and showed that the frequency of most of the tested markers was similar in the different groups of mothers and their neonates (**Figure 3A** and **Supplementary Figure 4**). However, production of estradiol was inversely correlated with NKG2A and NKp30, but directly correlated with NKG2C (**Figure 3B**), a marker overexpressed in several infections, including SARS-CoV-2 in patients with severe forms (13). Of note, no other phenotypic NK cell markers in mothers with ongoing infection and their neonates were correlated with estradiol production (**Supplementary Figure 5**). Furthermore, a significant decrease of NKp46 was observed in symptomatic patients (p = 0.0221), as compared to other patients with ongoing infection (**Supplementary Figure 3A**). Additionally, in neonates from mothers infected with SARS-CoV-2, expression of DNAX accessory molecule 1 (DNAM-1) was significantly increased, as compared to healthy pregnant women (**Figure 3C**). DNAM-1 is an activating receptor. Its ligands, belonging to the nectin family, are receptors for several viruses that mediate both the viral particle attack and entry, allowing the infection of cells of different origins (21).

Although the phenotypic profile of NK cells was only slightly impacted in mothers with ongoing infection and their neonates, these data reveal an important correlation between several key NK cell markers and estradiol.

### Impact of COVID-19 on the NK cell response in pregnant women and their neonates

**Figure 4:**
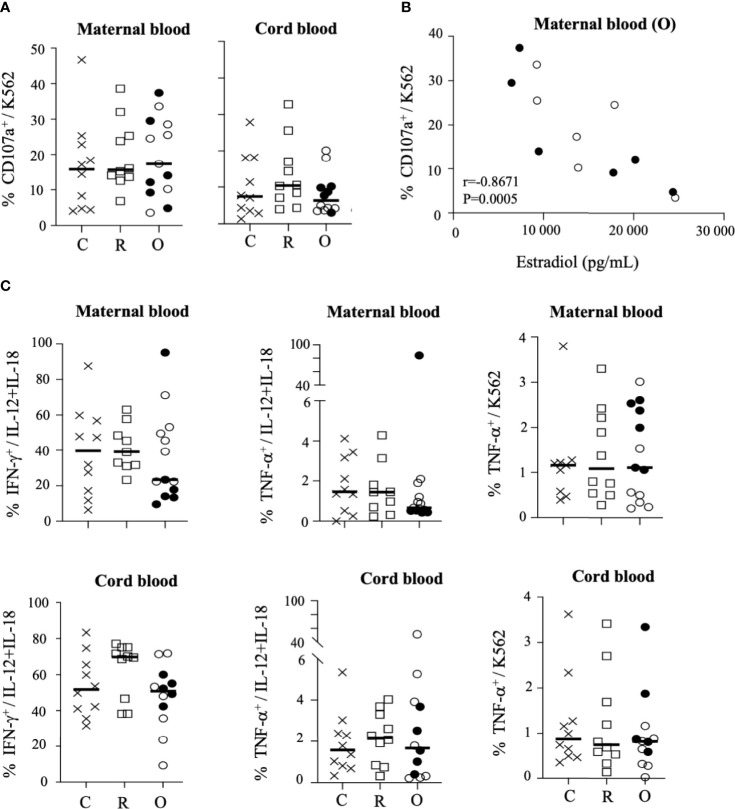
Degranulation and production of cytokines by CD3^-^CD56^+^ NK cells. **(A)** Degranulation of NK cells from mothers (maternal blood) and their neonates (cord blood), measured by cell surface expression of CD107a, in the presence of standard K562 target cells (ratio 1:1). **(B)** Correlation between CD107a and the production of estradiol in mothers (maternal blood) with ongoing infection (O: circles). **(C)** Intracellular production of IFN-γ and TNF-α in untreated cells (UT), after IL-12+IL-18 overnight stimulation (+IL-12/IL-18) or in the presence of standard K562 target cells (ratio 1:1). Data are shown NK cells of mothers (maternal blood) and their neonates (cord blood) from healthy controls (N= 10; C: crosses), recovered patients (N= 10; R: squares), and patients with ongoing infection (N=13; O: circles). Open circles represent the asymptomatic patients and closed circles the symptomatic patients. Black lines represent the median.

We next assessed the overall functional ability of NK cells. One of the main functions of these cells is their capacity to degranulate, measured by the cell surface expression of CD107a. Before stimulation, a low level of CD107a expression was detected in NK cells from SARS-CoV-2-infected patients (mothers and their neonates), similar to that observed in samples from healthy individuals (**Supplementary Figure 6**). In the presence of HLA class I-negative K562-sensitive target cells, the level of degranulation increased similarly in mothers and their neonates, unrelated to viral status (**Figure 4A**). However, in women with ongoing infection, the level of CD107a expression was very significantly correlated with estradiol (r = −0.8671; p = 0.0005) (**Figure 4B**).

We also investigated the ability of NK cells to synthesize and release IFN-γ and TNF-α. In the absence of stimulation, production of both cytokines was close to baseline in NK cells of all groups of mothers and their neonates (**Supplementary Figure 6**). Following overnight stimulation with IL-12 plus IL-18, IFN-γ production by maternal NK cells was strongly increased in healthy donors and recovered patients, but much less in mothers with ongoing infection, and more especially in symptomatic patients (**Figure 4C** and **Supplementary Figure 3A**). In contrast, in neonates the production of IFN-γ by NK cells was similar in all groups (**Figure 4C**). Of note, a pattern similar to that of IFN-γ was observed for the production of TNF-α by NK cells of mothers and their neonates (**Figure 4C**). Together, these data suggest a specific impairment of cytokine production by NK cells in symptomatic infected patients. However, none of these functional markers in mothers with ongoing infection and their neonates were correlated with estradiol production (**Supplementary Figure 5**). Unexpectedly, in the maternal NK cells of the symptomatic infected woman with fetal demise (p34 patient), we observed very high production of IFN-γ (95.1% vs. 16.6 ± 6.5% in other symptomatic patients) and TNF-α (83.9% vs. 0.6 ± 0.1% in other symptomatic patients) by NK cells after stimulation with IL-12 + IL-18 (**Figure 4C** and **Supplementary Figure 1**), whereas the production of IFN-γ and TNF-α in the plasma of this patient was similar to that observed in other individuals (**Supplementary Figure 7**), suggesting that overexpression of cytokines was specific to NK cells. However, in the absence of other similar cases, we were unable to establish if this unique immune imprinting was related to the fatal outcome observed in this patient.

Altogether, these data reveal that the functional capacities of NK cells from mothers with ongoing infection and their neonates were poorly increased after stimulation, except in the patient with severe COVID-19 (p34 patient).

### Characteristics of NK cells from pregnant women and their neonates in COVID-19 are associated with production of specific cytokines

**Figure 5:**
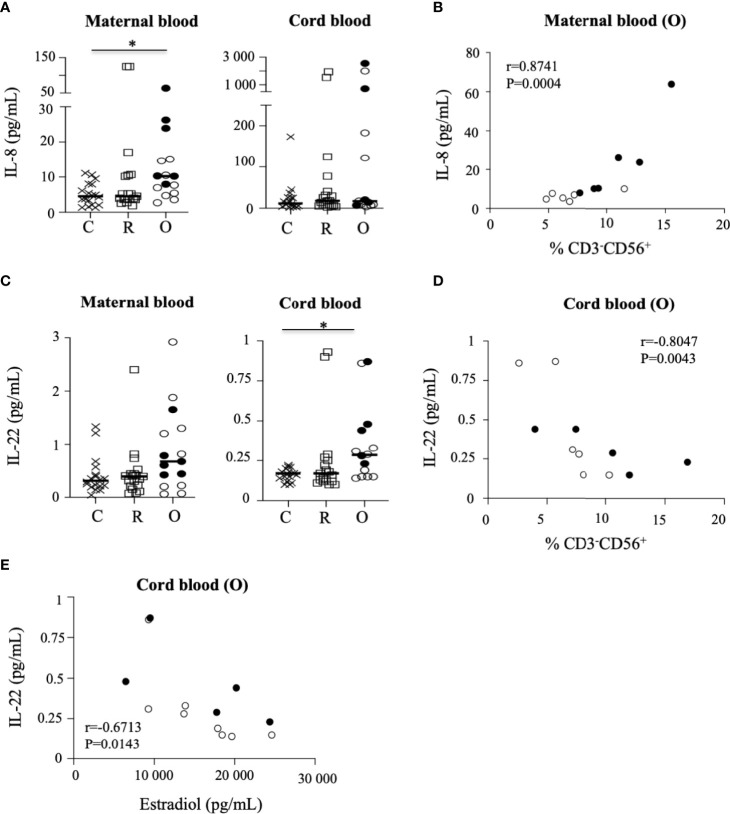
Production of cytokines in the sera of mothers and their neonates. **(A)** Production of IL-8 in the sera of mothers (maternal blood) and their neonates (cord blood). **(B)** Correlation between IL-8 production and the frequency of NK cells in mothers (maternal blood) with ongoing infection (O: circles). **(C)** Production of IL-22 in the sera of mothers (maternal blood) and their neonates (cord blood). **(D)** Correlation between IL-22 production and the frequency of NK cells in neonates born to mothers with ongoing infection (cord blood (O)). **(E)** Correlation between production of IL-22 production in neonates (cord blood) born to mothers with ongoing infection (cord blood (O)) and the production of estradiol in mothers with ongoing infection. Data are shown for healthy controls (N= 18; C: crosses), recovered patients (N= 19; R: squares), and patients with ongoing infection (N=15; O: circles). Open circles represent the asymptomatic patients and closed circles the symptomatic patients. Black lines represent the median.

In the general population, infection with SARS-CoV-2 is generally associated with the elevation of proinflammatory cytokines (22). Hence, we next determined the cytokine response in mothers and neonates by measuring the concentrations of cytokines in maternal and cord blood plasma using a multiplex assay. Plasma samples from non-infected and SARS-CoV-2-infected women were indistinguishable according to the production of IL-1β, IL-4, IL-5, and IL-6, and of IL-10, for which very low levels were observed (**Supplementary Figure 7**). In contrast, the production of IL-8 (p = 0.0277) and IL-10 (p = 0.0449) was significantly increased in patients with ongoing infection, as compared to recovered patients and healthy donors (**Figure 5A** and **Supplementary Figure 7**), as reported previously (19). Importantly, the production of IL-8 was also very significantly correlated with the frequency of maternal NK cells in patients with ongoing infection (r = 0.8741; p = 0.0004) (**Figure 5B**).

When assessing neonatal cytokine levels in cord blood, most of the cytokines tested were mostly undetectable in samples from non-infected and infected mothers (**Supplementary Figure 7**), except for IL-22, the production of which was significantly increased in neonates of patients with ongoing infection (p = 0.0497) (**Figure 5C**). Although the level quantified remained low, IL-22 production in cord blood samples from patients with ongoing infection was significantly correlated with the frequency of NK cells in neonates (r = −0.8047; p = 0.0043) (**Figure 5D**) and the production of estradiol (r = −0.6713; p = 0.0143) (**Figure 5E**).

## Discussion

The present study provides novel insights into the role of NK cells in the pathophysiology of pregnant women infected by SARS-CoV-2 and their neonates early after birth. As previously observed, the absolute value of NK cells from pregnant women was significantly decreased in COVID-19 (19), while the frequency of activated HLA-DR^+^ NK cells in pregnant women infected with SARS-CoV-2, and their neonates, was not increased and remained similar to that observed in healthy pregnant women controls. The absence of strong peripheral NK cell activation, generally observed in the general population infected with SARS-CoV-2, could be explained by assuming that to tolerate the fetus, the maternal immune system is modified during pregnancy (13, 23–25).

According to the fine-tuned equilibrium between inhibitory and activating NK cell receptors, early reports suggested that inhibitory checkpoint receptors on NK cells might contribute to the dysfunctional status of COVID-19-associated NK cells in the general population, although conflicting data have been reported; Demaria et al. (26) detected a higher level of NKG2A in COVID-19-associated NK cells from the peripheral blood and bronchoalveolar lavage fluid, whereas Maucourant et al. (13) reported an increase in NKG2C^+^ adaptive NK cells in patients with severe disease, as previously described in other acute infections (27–29). In pregnant women close to delivery, the phenotype of maternal NK cells was similar in infected patients and healthy controls, suggesting limited NK cell derangement in pregnant women infected by the SARS-CoV-2. Unexpectedly, expression of NKp30 and NKG2A in NK cells from pregnant women with ongoing infection was negatively correlated with estradiol in the serum, whereas NKG2C expression was positively correlated with estradiol, suggesting a role of estradiol in NK cell activity. Several studies have shown that the immune response to viruses can be modified by estradiol, as shown during SARS-CoV and MERS (Middle East respiratory syndrome coronavirus) epidemics. Furthermore, animal studies have shown that the use of estradiol receptor antagonists favors SARS-CoV infection (30). In COVID-19, estradiol could be linked to different mechanisms of action such as the reduction of expression of ACE-2 (angiotensin-converting enzyme 2), a SARS-CoV-2 receptor on target cells, and/or anti-inflammatory and immunomodulation effects (31). Consistently, the frequency of NK cells from pregnant women with ongoing infection able to degranulate is strongly inversely correlated with estradiol in the serum of normal pregnant women (32). Furthermore, a negative correlation between NK cell cytotoxicity and increasing doses of estradiol has been observed (32).

Altogether, these data suggest that high doses of estradiol could contribute to pregnancy-associated NK cell suppression, and indirectly to the control of SARS-CoV-2 infection, highlighting the key role of estradiol in the phenotypic and functional control of maternal NK cells during SARS-CoV-2 infection.

We also observed that the level of IFN-γ and TNF-α in NK cells was decreased, more especially in symptomatic patients with ongoing infection, as previously observed in individuals with severe disease. It has been suggested that the decrease of cytokine production could be linked to the ability of COVID-19-associated NK cells to reduce viral protein levels in SARS-CoV-2-infected cells (33). This effect was not observed in the plasma of pregnant patients infected by SARS-CoV-2, suggesting a specific control of cytokine production by NK cells from pregnant women. On the contrary, very high levels of IFN-γ and TNF-α were produced by NK cells from the symptomatic p34 patient, suggesting a possible role in the fetal demise. However, in the absence of other similar clinical cases we cannot conclude their participation in the cytokine storm observed in patients with severe disease (34, 35), which could possibly mediate pregnancy complications and fetal developmental pathology (36, 37). In accordance with Garcia-Flores (19), we also observed that pregnant women mount a specific inflammatory response to SARS-CoV-2, characterized by significantly increased levels of IL-8 and IL-10, also described in non-pregnant individuals with SARS-CoV-2 infection (38). One explanation for the seemingly paradoxical observation of concurrently elevated IL-10 and IL-8 levels is the ability of IL-10 to act as a proinflammatory and immune-stimulatory molecule in certain contexts (39). Interestingly, we found that IL-8 production was significantly correlated with frequency of NK cells from patients with ongoing infection. IL-8 is a canonical proinflammatory cytokine whose primary function is neutrophil recruitment to sites of injury (40). Recent studies have proposed that IL-8 can serve as a biomarker for the prediction of disease severity of patients infected with SARS-CoV-2 (41).

Next, we determined the effect of maternal infection with SARS-CoV-2 on NK cells in neonates; the hypothesis was that *in utero* exposure to infection and/or maternal inflammation could affect the NK cell system of the neonate and subsequently the response to the infection and possibly the future development of the baby. Consistently, we observed that both the absolute value and frequency of NK cells of neonates born to women with ongoing infection were significantly decreased. Despite the fact that a decrease in peripheral NK cells was previously observed in the general population, including in cases of severe COVID-19 (12, 14) but not in pregnant women, it is unclear whether this decrease in the number of NK cells in newborn babies was due to NK cell trafficking to infected tissues or cell death in the periphery. More importantly, the frequency of cord blood NK cells from mothers with ongoing infection was directly correlated with estradiol, suggesting that estradiol could play a key role in features of NK cells from women and their neonates. Further studies to investigate the underlying mechanism of estradiol in the maternal–fetal interaction NK cell responses triggered by SARS-CoV-2 should be performed.

In addition, we observed that the functional capacities of NK cells of neonates from COVID-19^+^ women were weak. This could be related to the absence of direct infection by SARS-CoV-2 at any point during gestation (42) or to their immaturity. Consistently, NK cells from neonates displayed specific phenotypic features associated with a less mature stage of differentiation that differed from adult NK cells (7, 43, 44). However, NK cells of neonates born to mothers infected with SARS-CoV-2 were similar to those of healthy controls, except for DNAM-1, an activating receptor significantly increased in COVID-19 that promotes activation, proliferation, cytokine production, and cytotoxic activity in NK cells (45, 46). The role of DNAM-1 in the control of viral infections has been underlined by the absence of control of lymphocytic choriomeningitis virus infection in DNAM-1^−/−^ mice (47), the NK cell recognition of virus-infected cells during early infection (48), and the capacity of DNAM-1^+^ NK cells to produce high levels of inflammatory cytokines (49). Consistently, the production of IL-22 was significantly increased in the serum of neonates from women infected with SARS-CoV-2, inversely correlated with both the frequency of NK cells in neonates and the level of estradiol quantified in the serum of pregnant women. IL-22 is a member of the IL-10 family of cytokines implicated in antiviral immune responses, and more especially during pulmonary infection; like IL-10, it has the double function of suppressing or encouraging inflammation in various disease models (50), but its role in COVID-19 remains elusive.

Altogether, these data highlight that SARS-CoV-2 infection induces phenotypic and functional modulations of peripheral NK cells from mothers and their neonates in correlation with inflammation markers, and estradiol.

There are several limitations to our study, in particular the relatively small number of symptomatic infected individuals, and more particularly patients with severe forms of COVID-19, with regard to the unexpected data obtained with p34 patient in whom infection caused fetal death, in accordance with recent data (51). In order to have more homogeneous groups, the pregnant women were included over a short period of time to limit the potential problems associated with successive appearance of the different variants and the bias posed by vaccination. It is understood, however, that it would be interesting to be able to apprehend them in subsequent studies. Furthermore, we only studied peripheral blood and cord blood NK cells and were not able to sample lung-resident NK cells or decidual NK cells. We also had limited data reflecting the history of the pregnant and control women; thus, we cannot exclude that some unmeasured factor could have influenced the NK cell phenotype and the quality of the NK cell responses to COVID-19. Finally, although new clinical data were obtained 1 month postpartum for the majority of mothers and their neonates, long-term follow-up of the newborns in our study would establish if maternal exposure to SARS-CoV-2 has some long-lasting impact on NK cells in the child.

Although the mechanisms underpinning our observations require further investigations, it seems clear that NK cells are associated with important aspects of COVID-19 pathophysiology in pregnant women and their neonates.

## Data availability statement

The original contributions presented in the study are included in the article/**Supplementary Material**. Further inquiries can be directed to the corresponding author.

## Ethics statement

The MaterCov study was conducted in accordance with the principles of the Declaration of Helsinki, as well as French statutory and regulatory law, and received approval from the Institutional Review Board of Ouest V Rennes in December 2020 IRB number 2020-A03115-34, and declared as clinical trial (N°NCT04726111). The patients/participants provided their written informed consent to participate in this study.

## Author contributions

MC, JA, and VV designed the study. AH, MC, CD, and MV contributed to clinical and biological data acquisition. NT, OG, VM, and AR carried out the experimental work. VV, MC, NT, OG, and CD contributed to data acquisition, analysis and interpretation. VV and TK performed statistical analysis. JA and VV provided financial support. VV, MC, and CD wrote the manuscript. All authors contributed to reviewing the manuscript. All authors contributed to the article and approved the submitted version.

## Funding

This project has received internal funding from Foch Foundation and the Institut National de la Santé et de la Recherche Médicale (Inserm), France.

## Conflict of interest

The authors declare that the research was conducted in the absence of any commercial or financial relationships that could be construed as a potential conflict of interest.

## Publisher’s note

All claims expressed in this article are solely those of the authors and do not necessarily represent those of their affiliated organizations, or those of the publisher, the editors and the reviewers. Any product that may be evaluated in this article, or claim that may be made by its manufacturer, is not guaranteed or endorsed by the publisher.

## References

[1] Mathew D Giles JR Baxter AE Oldridge DA Greenplate AR Wu JE. Deep immune profiling of COVID-19 patients reveals distinct immunotypes with therapeutic implications. Science (2020) 369(6508):eabc8511. doi: 10.1126/science.abc8511 32669297 PMC7402624

[2] Ramsey PS Ramin KD. Pneumonia in pregnancy. Obstet Gynecol Clin North Am (2001) 28(3):553–69. doi: 10.1016/s0889-8545(05)70217-5 11512500

[3] Jafari M Pormohammad A Sheikh Neshin SA Ghorbani S Bose D Alimohammadi S. Clinical characteristics and outcomes of pregnant women with COVID-19 and comparison with control patients: A systematic review and meta-analysis. Rev Med Virol (2021) 31(5):1–16. doi: 10.1002/rmv.2208 PMC7883245 33387448

[4] Conde-Agudelo A Romero R. SARS-CoV-2 infection during pregnancy and risk of preeclampsia: A systematic review and meta-analysis. Am J Obstet Gynecol (2022) 226(1):68–89 e3. doi: 10.1016/j.ajog.2021.07.009 34302772 PMC8294655

[5] Hanna J Goldman-Wohl D Hamani Y Avraham I Greenfield C Natanson-Yaron S. Decidual NK cells regulate key developmental processes at the human fetal-maternal interface. Nat Med (2006) 12(9):1065–74. doi: 10.1038/nm1452 16892062

[6] Watanabe M Iwatani Y Kaneda T Hidaka Y Mitsuda N Morimoto Y. Changes in T, b, and NK lymphocyte subsets during and after normal pregnancy. Am J Reprod Immunol (1997) 37(5):368–77. doi: 10.1111/j.1600-0897.1997.tb00246.x 9196795

[7] Le Garff-Tavernier M Beziat V Decocq J Siguret V Gandjbakhch F Pautas E. Human NK cells display major phenotypic and functional changes over the life span. Aging Cell (2010) 9(4):527–35. doi: 10.1111/j.1474-9726.2010.00584.x 20477761

[8] Orange JS Ballas ZK. Natural killer cells in human health and disease. Clin Immunol (2006) 118(1):1–10. doi: 10.1016/j.clim.2005.10.011 16337194

[9] Vivier E Raulet DH Moretta A Caligiuri MA Zitvogel L Lanier LL. Innate or adaptive immunity? the example of natural killer cells. Science (2011) 331(6013):44–9. doi: 10.1126/science.1198687 PMC3089969 21212348

[10] Sun JC Lanier LL. NK cell development, homeostasis and function: Parallels with CD8(+) T cells. Nat Rev Immunol (2011) 11(10):645–57. doi: 10.1038/nri3044 PMC4408539 21869816

[11] Koehl U Toubert A Pittari G. Editorial: Tailoring NK cell receptor-ligand interactions: An art in evolution. Front Immunol (2018) 9:351. doi: 10.3389/fimmu.2018.00351 29535727 PMC5835124

[12] Zheng M Gao Y Liu S Sun D Yang F Zong L. Serum inflammatory factors are positively correlated with the production of specific antibodies in coronavirus disease 2019 patients. Cell Mol Immunol (2020) 17(11):1180–2. doi: 10.1038/s41423-020-00551-1 PMC7506822 32963357

[13] Maucourant C Filipovic I Ponzetta A Aleman S Cornillet M Hertwig L. Natural killer cell immunotypes related to COVID-19 disease severity. Sci Immunol (2020) 5(50):eabd6832. doi: 10.1126/sciimmunol.abd6832 32826343 PMC7665314

[14] Wilk AJ Rustagi A Zhao NQ Roque J Martinez-Colon GJ McKechnie JL. A single-cell atlas of the peripheral immune response in patients with severe COVID-19. Nat Med (2020) 26(7):1070–6. doi: 10.1038/s41591-020-0944-y PMC7382903 32514174

[15] Bortolotti D Gentili V Rizzo S Rotola A Rizzo R. SARS-CoV-2 spike 1 protein controls natural killer cell activation *via* the HLA-E/NKG2A pathway. Cells (2020) 9(9):1975. doi: 10.3390/cells9091975 PMC7563485 32859121

[16] Gee S Chandiramani M Seow J Pollock E Modestini C Das A. The legacy of maternal SARS-CoV-2 infection on the immunology of the neonate. Nat Immunol (2021) 22(12):1490–502. doi: 10.1038/s41590-021-01049-2 34616036

[17] Cossarizza A Chang HD Radbruch A Akdis M Andra I Annunziato F. Guidelines for the use of flow cytometry and cell sorting in immunological studies. Eur J Immunol (2017) 47(10):1584–797. doi: 10.1002/eji.201646632 PMC9165548 29023707

[18] Bennabi M Tarantino N Gaman A Scheid I Krishnamoorthy R Debre P. Persistence of dysfunctional natural killer cells in adults with high-functioning autism spectrum disorders: Stigma/Consequence of unresolved early infectious events? Mol Autism (2019) 10:22. doi: 10.1186/s13229-019-0269-1 31123562 PMC6521549

[19] Garcia-Flores V Romero R Xu Y Theis KR Arenas-Hernandez M Miller D. Maternal-fetal immune responses in pregnant women infected with SARS-CoV-2. Nat Commun (2022) 13(1):320. doi: 10.1038/s41467-021-27745-z 35042863 PMC8766450

[20] Schwane V Huynh-Tran VH Vollmers S Yakup VM Sauter J Schmidt AH. Distinct signatures in the receptor repertoire discriminate CD56Bright and CD56dim natural killer cells. Front Immunol (2020) 11:568927. doi: 10.3389/fimmu.2020.568927 33335526 PMC7736243

[21] Cifaldi L Doria M Cotugno N Zicari S Cancrini C Palma P. DNAM-1 activating receptor and its ligands: How do viruses affect NK cell-mediated immune surveillance during the various phases of infection? Int J Mol Sci (2019) 20(15):3715. doi: 10.3390/ijms20153715 PMC6695959 31366013

[22] Coomes EA Haghbayan H. Interleukin-6 in covid-19: A systematic review and meta-analysis. Rev Med Virol (2020) 30(6):1–9. doi: 10.1002/rmv.2141 PMC7460877 32845568

[23] Manickam C Sugawara S Reeves RK. Friends or foes? the knowns and unknowns of natural killer cell biology in COVID-19 and other coronaviruses in July 2020. PloS Pathog (2020) 16(8):e1008820. doi: 10.1371/journal.ppat.1008820 32845937 PMC7449465

[24] Ander SE Diamond MS Coyne CB. Immune responses at the maternal-fetal interface. Sci Immunol (2019) 4(31):eaat6114. doi: 10.1126/sciimmunol.aat6114 30635356 PMC6744611

[25] Yang F Zheng Q Jin L. Dynamic function and composition changes of immune cells during normal and pathological pregnancy at the maternal-fetal interface. Front Immunol (2019) 10:2317. doi: 10.3389/fimmu.2019.02317 31681264 PMC6813251

[26] Demaria O Carvelli J Batista L Thibult ML Morel A Andre P. Identification of druggable inhibitory immune checkpoints on natural killer cells in COVID-19. Cell Mol Immunol (2020) 17(9):995–7. doi: 10.1038/s41423-020-0493-9 PMC7327215 32612152

[27] Bjorkstrom NK Lindgren T Stoltz M Fauriat C Braun M Evander M. Rapid expansion and long-term persistence of elevated NK cell numbers in humans infected with hantavirus. J Exp Med (2011) 208(1):13–21. doi: 10.1084/jem.20100762 21173105 PMC3023129

[28] Maucourant C Nonato Queiroz GA Corneau A Leandro Gois L Meghraoui-Kheddar A Tarantino N. NK cell responses in zika virus infection are biased towards cytokine-mediated effector functions. J Immunol (2021) 207(5):1333–43. doi: 10.4049/jimmunol.2001180 34408012

[29] Petitdemange C Becquart P Wauquier N Beziat V Debre P Leroy EM. Unconventional repertoire profile is imprinted during acute chikungunya infection for natural killer cells polarization toward cytotoxicity. PloS Pathog (2011) 7(9):e1002268. doi: 10.1371/journal.ppat.1002268 21966274 PMC3178577

[30] Channappanavar R Fett C Mack M Ten Eyck PP Meyerholz DK Perlman S. Sex-based differences in susceptibility to severe acute respiratory syndrome coronavirus infection. J Immunol (2017) 198(10):4046–53. doi: 10.4049/jimmunol.1601896 PMC5450662 28373583

[31] Nakaya M Tachibana H Yamada K. Effect of estrogens on the interferon-gamma producing cell population of mouse splenocytes. Biosci Biotechnol Biochem (2006) 70(1):47–53. doi: 10.1271/bbb.70.47 16428820

[32] Gabrilovac J Zadjelovic J Osmak M Suchanek E Zupanovic Z Boranic M. NK cell activity and estrogen hormone levels during normal human pregnancy. Gynecol Obstet Invest (1988) 25(3):165–72. doi: 10.1159/000293766 3391426

[33] Bi J. NK cell dysfunction in patients with COVID-19. Cell Mol Immunol (2022) 19(2):127–9. doi: 10.1038/s41423-021-00825-2 PMC8755515 35022604

[34] Blanco-Melo D Nilsson-Payant BE Liu WC Uhl S Hoagland D Moller R. Imbalanced host response to SARS-CoV-2 drives development of COVID-19. Cell (2020) 181(5):1036–45 e9. doi: 10.1016/j.cell.2020.04.026 32416070 PMC7227586

[35] Chen G Wu D Guo W Cao Y Huang D Wang H. Clinical and immunological features of severe and moderate coronavirus disease 2019. J Clin Invest (2020) 130(5):2620–9. doi: 10.1172/JCI137244 PMC7190990 32217835

[36] Robertson SA Chin PY Femia JG Brown HM. Embryotoxic cytokines-potential roles in embryo loss and fetal programming. J Reprod Immunol (2018) 125:80–8. doi: 10.1016/j.jri.2017.12.003 29306096

[37] Yockey LJ Iwasaki A. Interferons and proinflammatory cytokines in pregnancy and fetal development. Immunity (2018) 49(3):397–412. doi: 10.1016/j.immuni.2018.07.017 30231982 PMC6152841

[38] Kunnumakkara AB Rana V Parama D Banik K Girisa S Henamayee S. COVID-19, cytokines, inflammation, and spices: How are they related? Life Sci (2021) 284:119201. doi: 10.1016/j.lfs.2021.119201 33607159 PMC7884924

[39] Islam H Chamberlain TC Mui AL Little JP. Elevated interleukin-10 levels in COVID-19: Potentiation of pro-inflammatory responses or impaired anti-inflammatory action? Front Immunol (2021) 12:677008. doi: 10.3309/fimmu.2021.677008 34234779 PMC8255680

[40] Azevedo MLV Zanchettin AC Vaz de Paula CB Motta Júnior JDS Malaquias MAS Raboni SM. Lung neutrophylic recruitment and IL-8/IL-17a tissue expression in COVID 19. Front Immunol (2021) 12:656350. doi: 10.3389/fimmu.2021.656350 33868301 PMC8044579

[41] Del Valle DM Kim-Schulze S Huang HH Beckmann ND Nirenberg S Wang B. An inflammatory cytokine signature predicts COVID-19 severity and survival. Nat Med (2020) 26(10):1636–43. doi: 10.1038/s41591-020-1051-9 PMC7869028 32839624

[42] Anderson J Thang CM Thanh LQ Dai VTT Phan VT Nhu BTH. Immune profiling of cord blood from preterm and term infants reveals distinct differences in pro-inflammatory responses. Front Immunol (2021) 12:777927. doi: 10.3389/fimmu.2021.777927 34790206 PMC8591285

[43] Dalle JH Menezes J Wagner E Blagdon M Champagne J Champagne MA. Characterization of cord blood natural killer cells: Implications for transplantation and neonatal infections. Pediatr Res (2005) 57(5 Pt 1):649–55. doi: 10.1203/01.PDR.0000156501.55431.20 15718362

[44] Sarvaria A Jawdat D Madrigal JA Saudemont A. Umbilical cord blood natural killer cells, their characteristics, and potential clinical applications. Front Immunol (2017) 8:329. doi: 10.3389/fimmu.2017.00329 28386260 PMC5362597

[45] Shibuya A Shibuya K. DNAM-1 versus TIGIT: Competitive roles in tumor immunity and inflammatory responses. Int Immunol (2021) 33(12):687–92. doi: 10.1093/intimm/dxab085 34694361

[46] Cifaldi L Doria M Cotugno N Zicari S Cancrini C Palma P. DNAM-1 activating receptor and its ligands: How do viruses affect the NK cell-mediated immune surveillance during the various phases of infection? Int J Mol Sci (2019) 20(15):3715. doi: 10.3390/ijms20153715 PMC6695959 31366013

[47] Welch MJ Teijaro JR Lewicki HA Colonna M Oldstone MB. CD8 T cell defect of TNF-alpha and IL-2 in DNAM-1 deficient mice delays clearance *In vivo* of a persistent virus infection. Virology (2012) 429(2):163–70. doi: 10.1016/j.virol.2012.04.006 PMC3571684 22579352

[48] Tomasec P Wang EC Davison AJ Vojtesek B Armstrong M Griffin C. Downregulation of natural killer cell-activating ligand CD155 by human cytomegalovirus UL141. Nat Immunol (2005) 6(2):181–8. doi: 10.1038/ni1156 PMC2844263 15640804

[49] Martinet L Ferrari De Andrade L Guillerey C Lee JS Liu J Souza-Fonseca-Guimaraes F. DNAM-1 expression marks an alternative program of NK cell maturation. Cell Rep (2015) 11(1):85–97. doi: 10.1016/j.celrep.2015.03.006 25818301

[50] Alcorn JF. IL-22 plays a critical role in maintaining epithelial integrity during pulmonary infection. Front Immunol (2020) 11:1160. doi: 10.3389/fimmu.2020.01160 32582219 PMC7296169

[51] Male V. SARS-CoV-2 infection and COVID-19 vaccination in pregnancy. Nat Rev Immunol (2021) 21(4):200–1. doi: 10.1038/s41577-021-00525-y PMC8931577 35304596

