# Supplementary material for: Plasticity of natural killer cells in pregnant patients infected with SARS-CoV-2 and their neonates during childbirth
Source: Front Immunol. 2022 Jul 15;13:893450. doi: 10.3389/fimmu.2022.893450 (PMC9335005; doi:10.3389/fimmu.2022.893450)
Supplement: Supplementary file 1 [file DataSheet_1.pdf]

Supplementary Table 1. List of markers used in the study

Marker	Clone	Fluorochromes	Companies
CD3	UCHT1	eFluor780	eBioscience
CD16	REA423	PerCP-Vio700	Miltenyi Biotec
CD56	REA196	PEVio770	Miltenyi Biotec
CD161	DX12	Fluorescein isothio-cyanate (FITC)	BD Pharmingen
CD69	TP1.55.3	ECD	Beckman Coulter
HLA-DR	I243	AlexaFluor700	BioLegend
CD57	NC1	Pacific Blue	Beckman Coulter
NKG2A (CD159a)	Z199	Phycoerythrin (PE)	Beckman Coulter
NKG2C (CD159c)	134591	Allophycocyanin (APC)	R&D System
NKG2D (CD314)	1D11	BV786	BD Optibuild
NKp30 (CD337)	P30-15	BV421	BD Horizon
NKp46 (CD335)	REA808	PeVio615	Miltenyi Biotec
KIR2DL1 (CD158a)	HP-3E4	Fluorescein isothio-cyanate (FITC)	BD Pharmingen
KIR2DL2/DL3 (CD158b/b2,j)	GL183	Phycoerythrin (PE)	Beckman Coulter
KIR3DL1 (CD158e1)	DX9	AlexaFluor700	Biolegend
ILT2 (CD85j)	GHI/75	Allophycocyanin (APC)	Miltenyi Biotec
DNAM-1 (CD226)	DX11	BV650	BD Optibuild
CD107a	H4A3	Fluorescein isothio-cyanate (FITC)	BD Pharmingen
IFN-γ	B27	AlexaFluor700	BD Pharmingen
TNF-α	Mab11	eFluor450	eBioscience
Viability		eFluor506	eBioscience

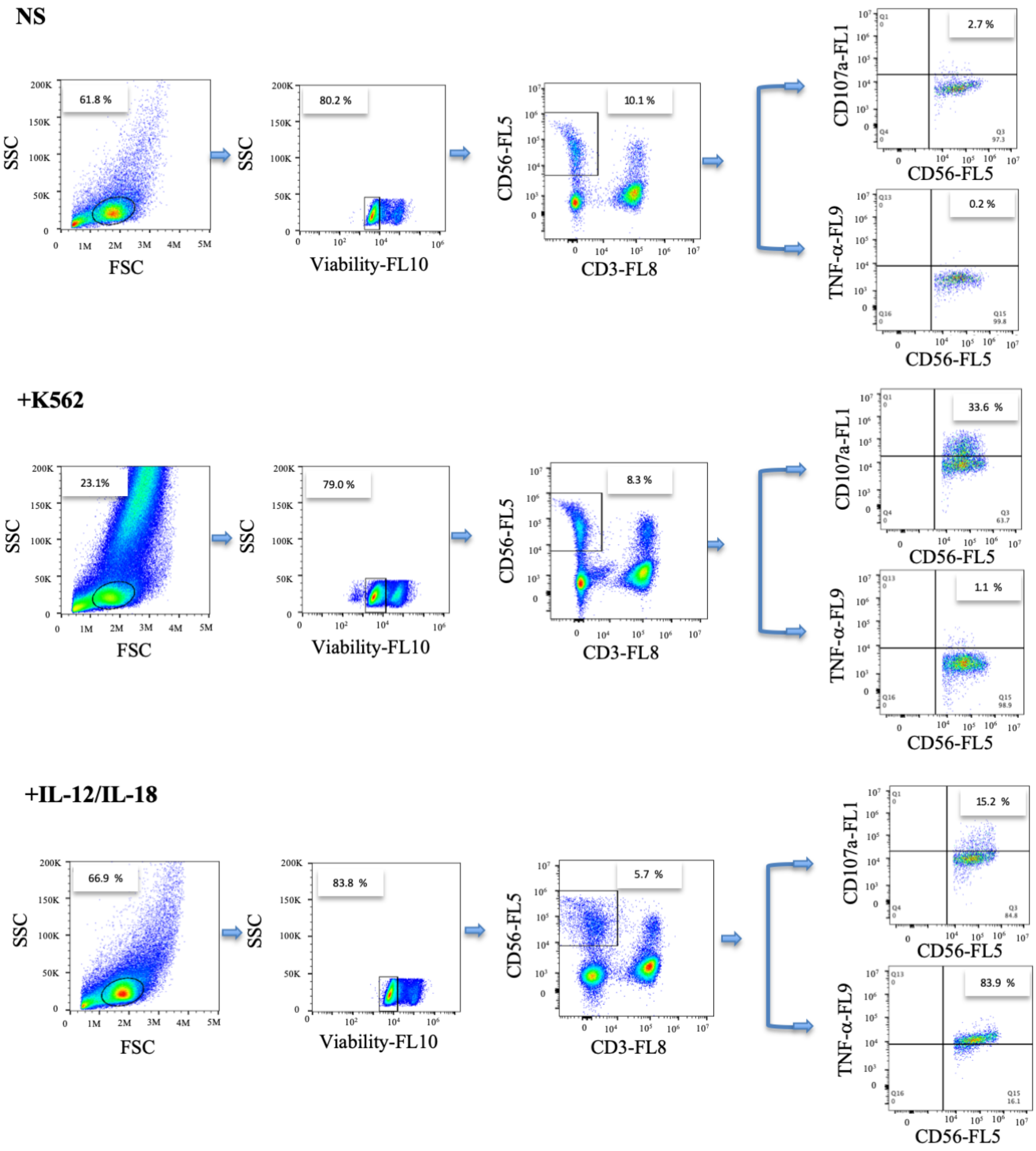

Supplementary Figure 1. Gating strategy

The $CD3^{-}CD56^{+}$ NK cells were identified within the alive lymphocyte population in the maternal blood and cord blood from SARS-CoV-2^{+} or control pregnant women. The data of degranulation and TNF-α production are shown for the p34 patient, without stimulation (NS), in the presence of K562 target cells (+K562), or after overnight pre-treatment with IL-12 plus IL-18 (+IL-12/IL-18).

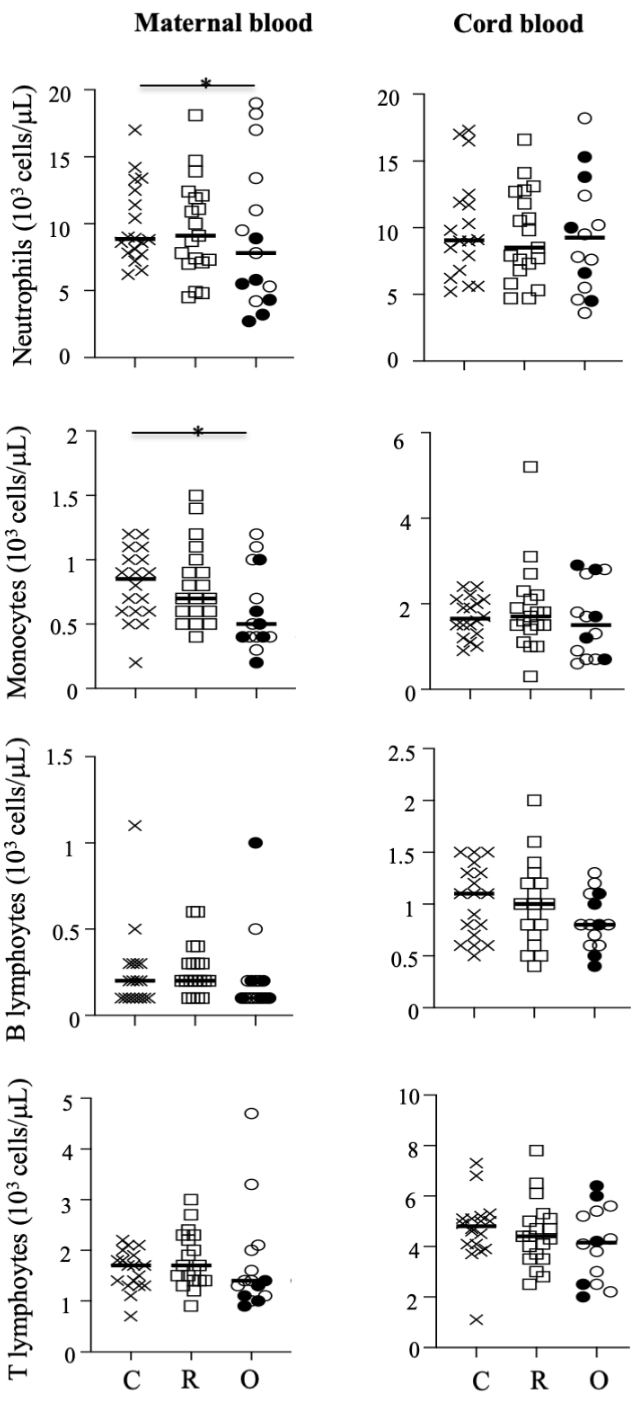

Supplementary Figure 2. Proportions of hematopoietic cell subsets.
Absolute values of neutrophils, monocytes, B lymphocytes, and T lymphocytes, measured by DXFlex (Beckman-Coulter) in the peripheral blood of mother (maternal blood) and their neonates (cord blood). Data are shown for healthy controls (N= 18; C: crosses), recovered patients (N= 19; R: squares), and patients with ongoing infection (N=15; O: circles). Open circles represent the asymptomatic patients and closed circles the symptomatic patients. Black lines represent the median. *$p < 0.05$.

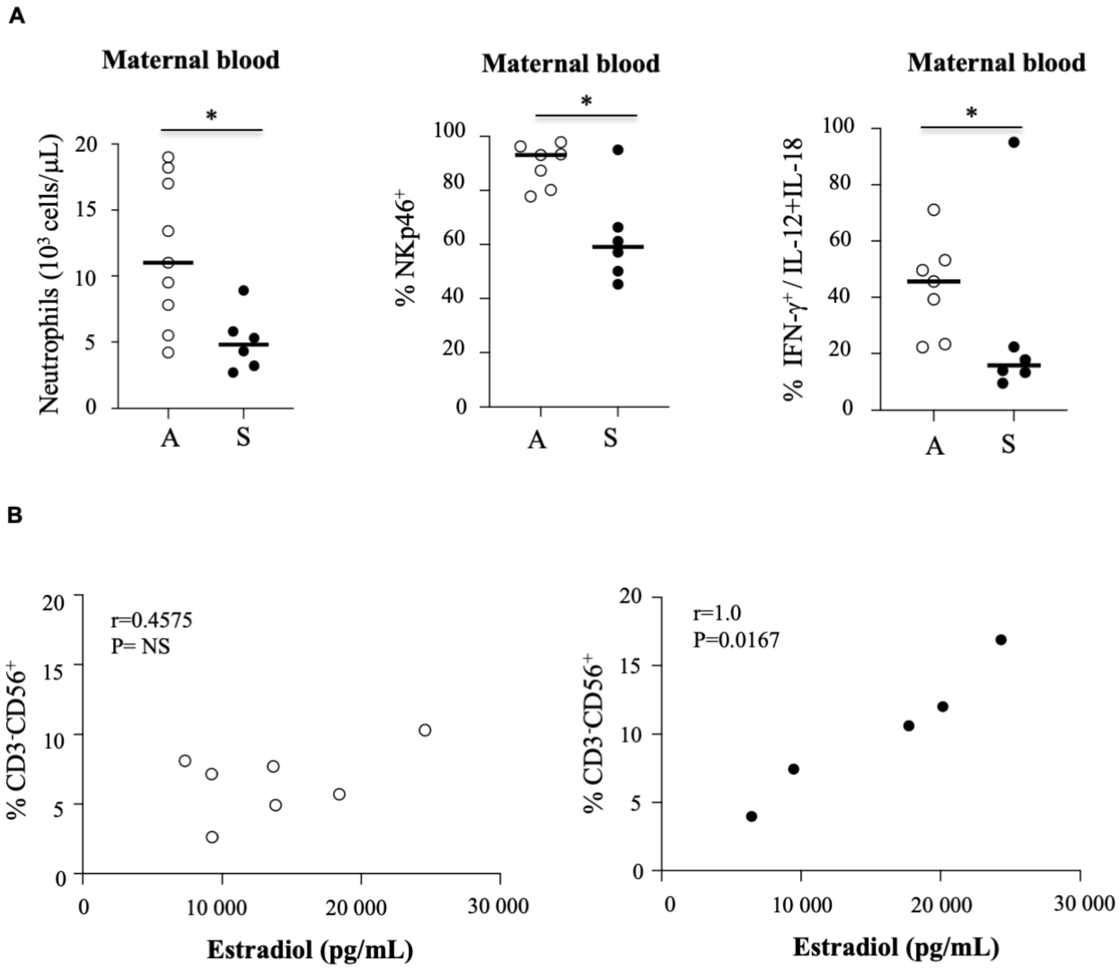

Supplementary Figure 3. Comparison between symptomatic and asymptomatic patients. **(A)** Analysis of absolute value of neutrophils as well as frequency of NKp46 and IFN-g production by NK cells from maternal blood of asymptomatic (open circles) and symptomatic (closed circles) patients with ongoing infection. Black lines represent the median. Statistical analysis was performed with Mann Whitney U-test. (**B)** Correlation between the production of estradiol in mothers and the frequency of $CD3^{-}CD56^{+}$ NK cells in neonates (cord blood) born to mothers with asymptomatic (open circles) or symptomatic (closed circles) ongoing infection. NS: non-significant.

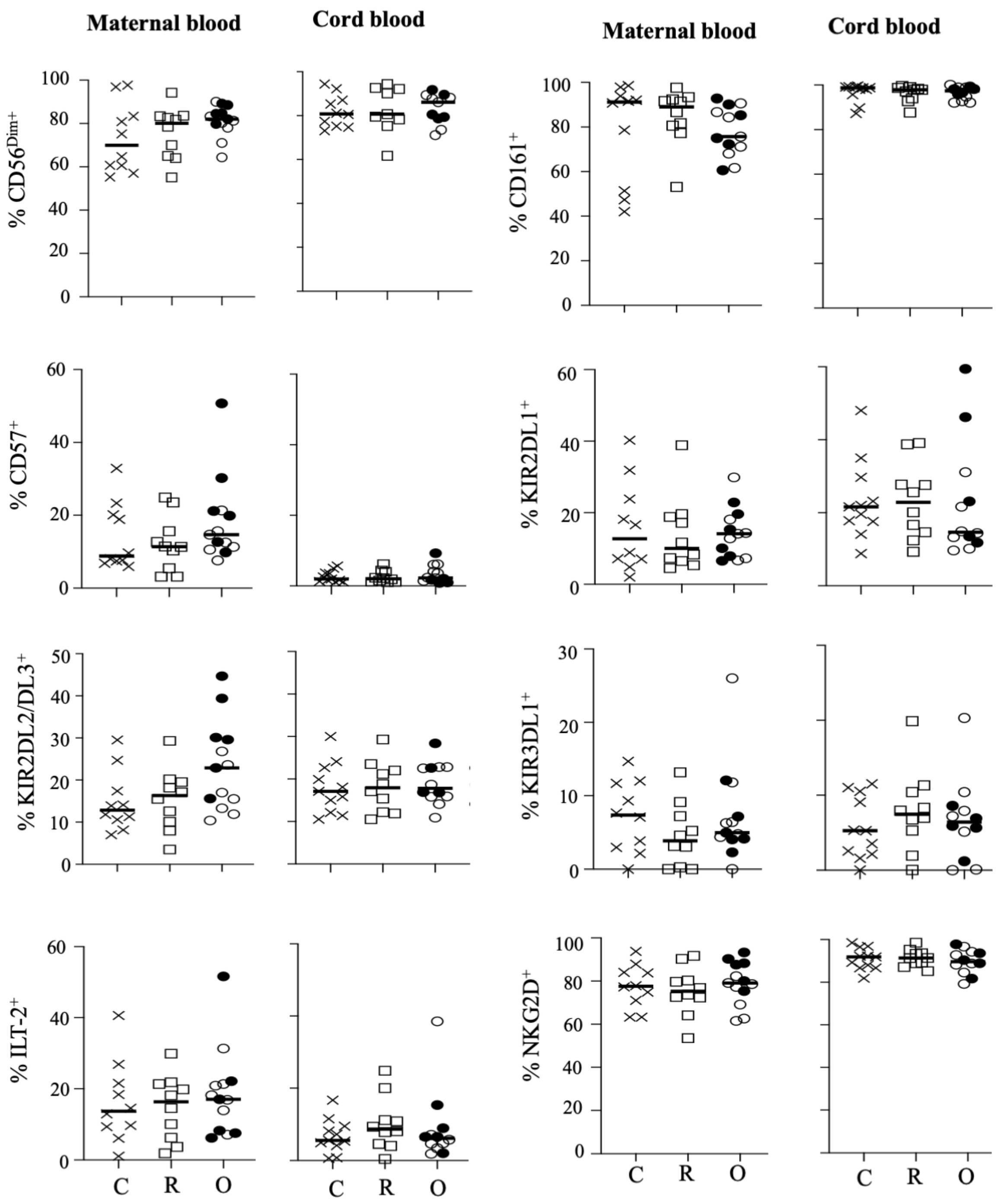

Supplementary Figure 4. Supplementary data on the phenotypic markers on NK cells. Frequency of CD56^{dim} on lymphocytic cells as well as CD161, CD57, inhibitory KIR, ILT-2, and NKG2D on NK cells gated on CD3^{-}CD56^{+} NK cells from mothers (maternal blood) and their neonates (cord blood). Data are shown for healthy controls (N= 10; C: crosses), recovered patients (N= 10; R: squares), and patients with ongoing infection (N=13; O: circles). Open circles represent the asymptomatic patients and closed circles the symptomatic patients. Black lines represent the median.

Maternal blood

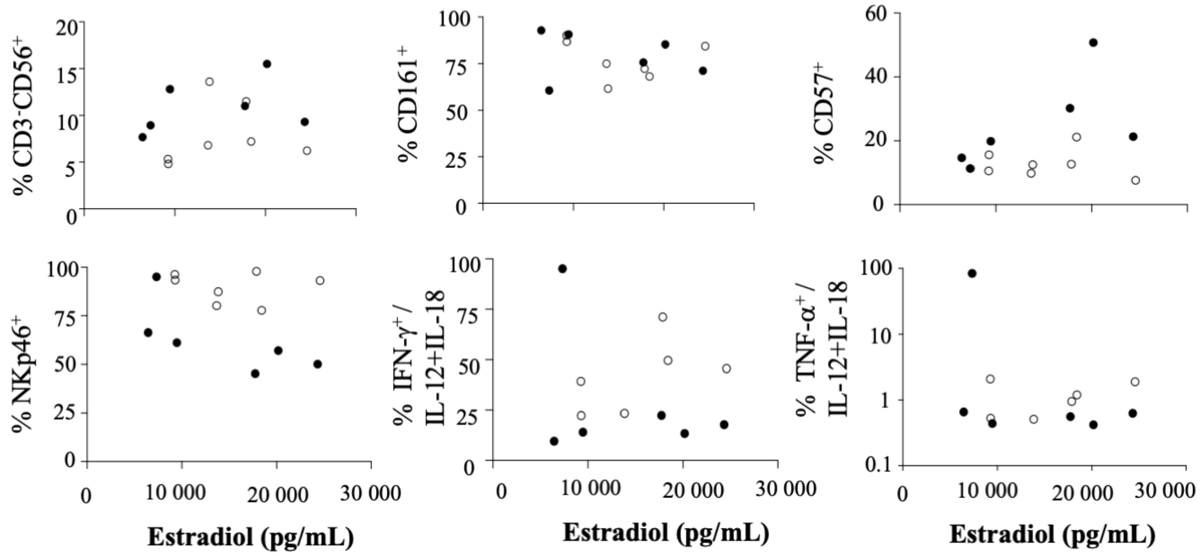

Cord blood

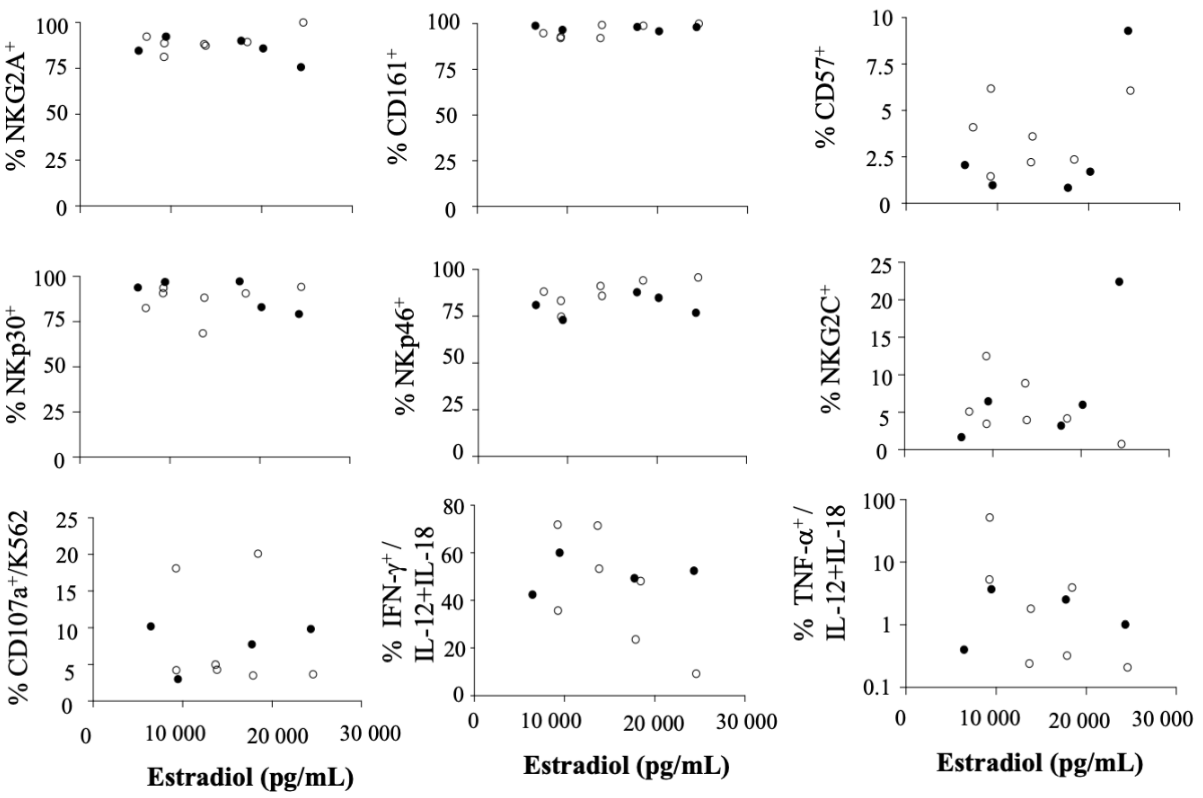

Supplementary Figure 5. Supplementary correlations between estradiol production and NK-cell markers

Data are shown for $CD3^{-}CD56^{+}$ NK cells from mothers (maternal blood) with ongoing infection and their neonates (Cord blood). Open circles represent the asymptomatic patients and closed circles the symptomatic patients.

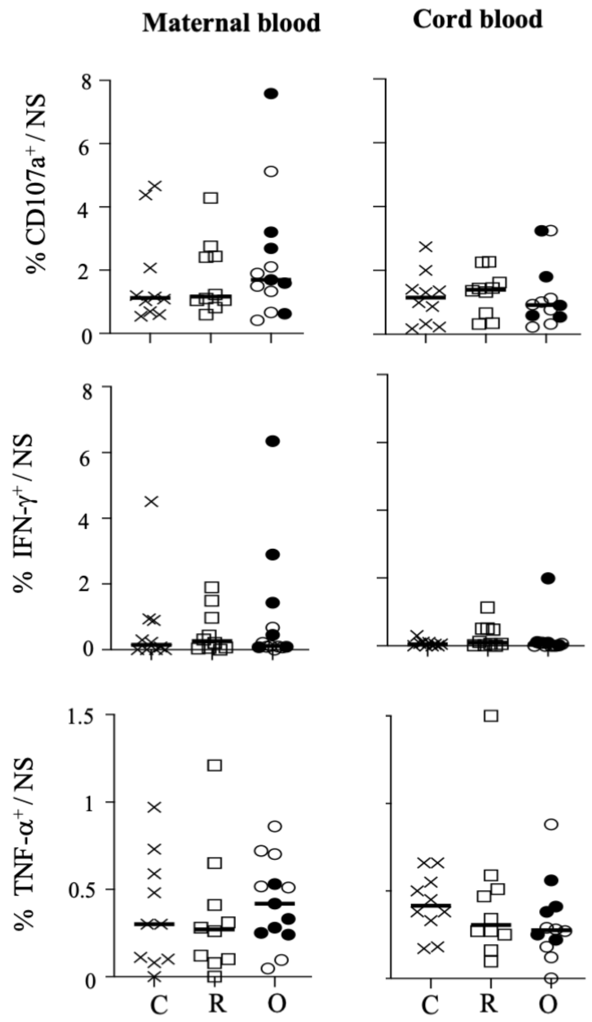

Supplementary Figure 6. Supplementary data of polyfunctional assays
Degranulation and production of IFN-γ and TNF-α by untreated (NS) $CD3^{-}CD56^{+}$ NK cells from mothers (maternal blood) and their neonates (cord blood). Data are shown for healthy controls (N= 10; C: crosses), recovered patients (N= 10; R: squares), and patients with ongoing infection (N=13; O: circles). Open circles represent the asymptomatic patients and closed circles the symptomatic patients. Black lines represent the median.

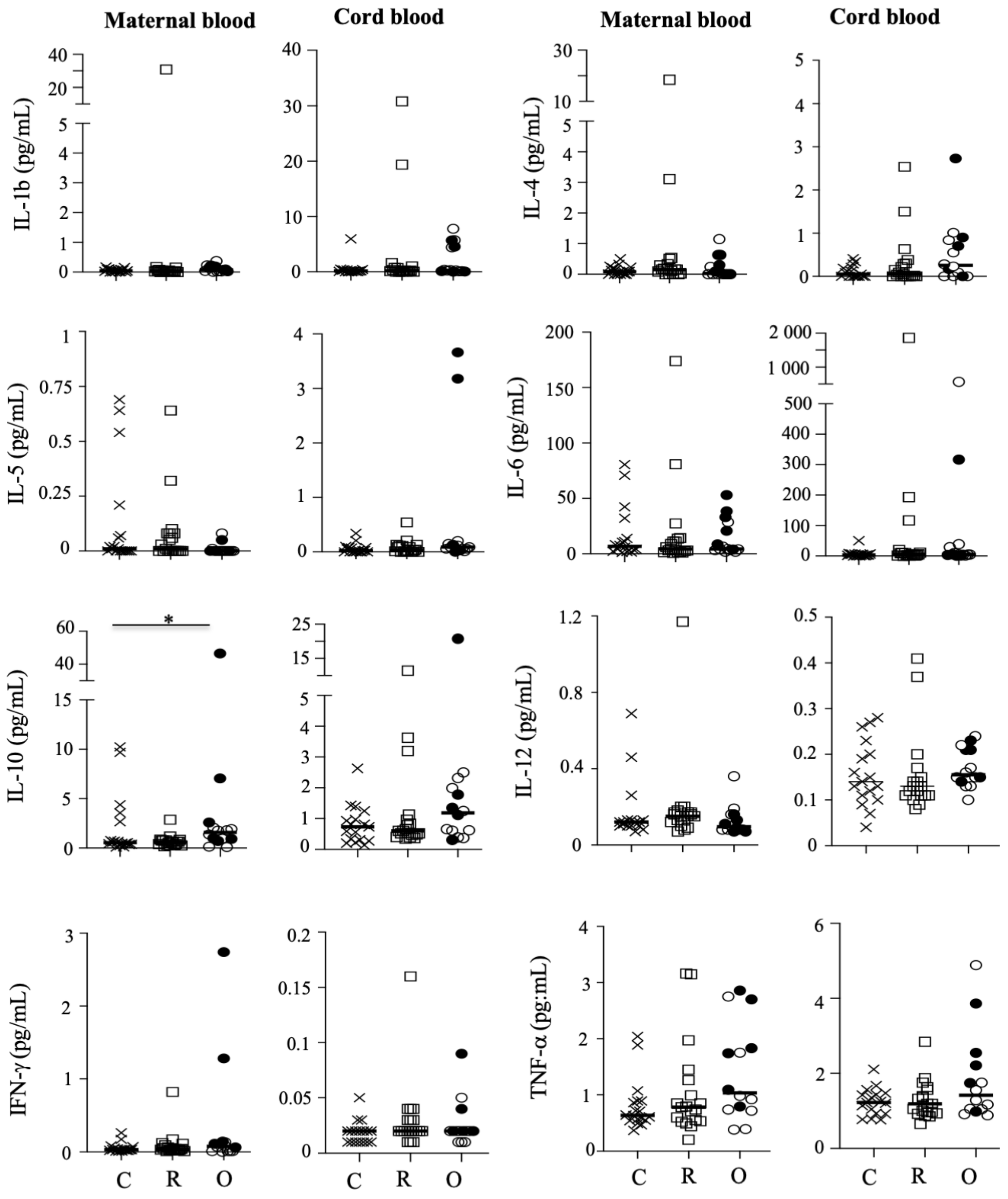

Supplementary Figure 7. Supplementary data on the production of cytokines in the sera. Cytokine production in the sera of mothers (maternal blood) and their neonates (cord blood). Data are shown for healthy controls (N= 18; C: crosses), recovered patients (N= 19; R: squares), and patients with ongoing infection (N=15; O: circles). Open circles represent the asymptomatic patients and closed circles the symptomatic patients. Black lines represent the median.
